# Rat Aquaporin-5 Is pH-Gated Induced by Phosphorylation and Is Implicated in Oxidative Stress

**DOI:** 10.3390/ijms17122090

**Published:** 2016-12-13

**Authors:** Claudia Rodrigues, Andreia Filipa Mósca, Ana Paula Martins, Tatiana Nobre, Catarina Prista, Fernando Antunes, Ana Cipak Gasparovic, Graça Soveral

**Affiliations:** 1 Research Institute for Medicines (iMed.ULisboa), Faculty of Pharmacy, Universidade de Lisboa, 1649-003 Lisboa, Portugal; crodrigues@ff.ulisboa.pt (C.R.); andreiafbm@ff.ulisboa.pt (A.F.M.); martinsap@ff.ulisboa.pt (A.P.M.); t.nobre@campus.ul.pt (T.N.); 2 Department of Biochemistry and Human Biology, Faculty of Pharmacy, Universidade de Lisboa, 1649-003 Lisboa, Portugal; 3 Linking Landscape, Environment, Agriculture and Food, Instituto Superior de Agronomia, Universidade de Lisboa, 1349-017 Lisboa, Portugal; cprista@isa.ulisboa.pt; 4 Centro de Química e Bioquímica e Departamento de Química e Bioquímica, Faculdade de Ciências, Universidade de Lisboa, 1749-016 Lisboa, Portugal; fantunes@fc.ul.pt; 5 Rudjer Boskovic Institute, HR 10000 Zagreb, Croatia; Ana.Cipak.Gasparovic@irb.hr

**Keywords:** aquaporin, yeast, permeability, phosphorylation, pH gating, reactive oxygen species, hydrogen peroxide, oxidative stress

## Abstract

Aquaporin-5 (AQP5) is a membrane water channel widely distributed in human tissues that was found up-regulated in different tumors and considered implicated in carcinogenesis in different organs and systems. Despite its wide distribution pattern and physiological importance, AQP5 short-term regulation was not reported and mechanisms underlying its involvement in cancer are not well defined. In this work, we expressed rat AQP5 in yeast and investigated mechanisms of gating, as well as AQP5’s ability to facilitate H_2_O_2_ plasma membrane diffusion. We found that AQP5 can be gated by extracellular pH in a phosphorylation-dependent manner, with higher activity at physiological pH 7.4. Moreover, similar to other mammalian AQPs, AQP5 is able to increase extracellular H_2_O_2_ influx and to affect oxidative cell response with dual effects: whereas in acute oxidative stress conditions AQP5 induces an initial higher sensitivity, in chronic stress AQP5 expressing cells show improved cell survival and resistance. Our findings support the involvement of AQP5 in oxidative stress and suggest AQP5 modulation by phosphorylation as a novel tool for therapeutics.

## 1. Introduction

Aquaporins (AQPs) are a family of highly conserved transmembrane channels that transport water and, in some cases, small solutes such as glycerol, driven by osmotic or solute gradients [1,2]. The 13 known mammalian isoforms (AQP0–12) are differentially expressed in several tissues/organs and show different permeability, structural features, and cellular localization. Importantly, these proteins are involved in many biological functions including transepithelial fluid transport, brain edema, neuroexcitation, cell migration, adhesion, proliferation, differentiation, and metabolism [3,4]. Their numerous roles in physiology make these proteins essential for health, suggesting that modulation of AQP’s function or expression could have therapeutic potential in edema, cancer, obesity, brain injury, glaucoma, and several other conditions [5].

Besides being widely distributed among the human body, AQP5 was found expressed in salivary and lacrimal glands and showed to play a major role in saliva secretion [6]. AQP5 transport defect was also associated with Sjögren’s syndrome, a chronic autoimmune disease that destroys the salivary and lacrimal glands [6]. More recently, AQP5 gained attention due its potential implication in carcinogenesis in different organs and systems [7]. AQP5 was found overexpressed in cancer cells and tumor tissues, strongly suggesting that it may be implicated in tumor formation by contributing to cell differentiation and migration through mechanisms involving AQP5 interplay with intracellular signaling transduction pathways. In tumors, the cAMP-dependent phosphorylation of AQP5 by PKA activates the RAS/Mitogen-activated protein kinases (MAPK) pathway involved in cell proliferation and survival [8,9,10]. In addition to AQP5 interaction with oncogenes, its function as a water channel was proposed to be involved in cell migration, since AQP5 expression may facilitate changes in cell volume and shape that are crucial for migration [11].

Recently, permeation of hydrogen peroxide (H_2_O_2_) by some AQPs revealed that these channels could influence regulatory complex signaling pathways involved in pathological states. It is now well established that reactive oxygen species (ROS), particularly H_2_O_2_, participate in cell signaling transduction pathways affecting cellular growth and proliferation mechanisms involved in cancer development [12,13].

Although it is generally assumed that H_2_O_2_ is highly diffusible across the membrane lipid bilayer, it was shown that actually the membrane lipid bilayer slows down permeation of H_2_O_2_, leading to the formation of gradients [14]. This barrier, however, may be overcome by the presence of some AQPs recently shown to be capable of transporting extracellular H_2_O_2_ into the cells [15]. In mammalian cells, AQP3, AQP8, and AQP9 were shown to mediate H_2_O_2_ membrane transport [16,17,18], which may be used for intracellular signaling in cancer cells [19]. Consequently, due to its involvement in cancer progression, we anticipated that AQP5 might also be able to facilitate H_2_O_2_ permeation and have a role in cell oxidative stress response explaining, at least in part, its overexpression in cancer tissues.

Interestingly, a recent study showed that AQP5 membrane abundance is regulated by phosphorylation [20], and in fact, the contrasting phosphorylation status between cancer and normal tissues suggests that AQP5 role in tumorigenesis is related with its phosphorylation [7]. Furthermore, gating mechanisms that induce a change in the 3D-channel structure and consequently affect its transport activity [2] have been described for several eukaryotic AQPs [21,22,23,24], among which pH and phosphorylation represent a short-term regulatory mechanism commonly used by several AQP family members [24,25]. However, besides its post-translational modification by phosphorylation, gating mechanisms for AQP5 channel activity regulation have not been reported so far.

An important tool to investigate AQP’s function and regulation is the yeast heterologous expression system [2]. Yeast cells lacking endogenous AQPs have been used to detect water transport capacity of mammalian [26,27] and plant [28,29] aquaporins and recently we used this system to characterize the pH gating of AQP3 [27].

In the current work, we cloned rat AQP5 in the yeast *Saccharomyces cerevisiae* and investigated its channel activity regulation by external pH and phosphorylation. We observed that AQP5 does not change its activity by external acidification, but phosphorylation makes the AQP5 channel prone to pH sensing. Moreover, AQP5 is able to modulate H_2_O_2_ transport through the plasma membrane and this feature interferes with oxidative cell response with dual effects: acute oxidative stress induces an initial higher sensitivity while long-term exposure and chronic stress conditions increase cell survival and resistance to the oxidative stress insult.

Thus, the current findings support a direct role of AQP5 in cancer development by mediating H_2_O_2_ membrane permeation, affecting redox signaling, and influencing signaling transduction pathways involved in tumorigenesis.

## 2. Results

### 2.1. Subcellular Localization and Water Permeability of Rat AQP5 Expressed in Yeast

**Figure 1:**
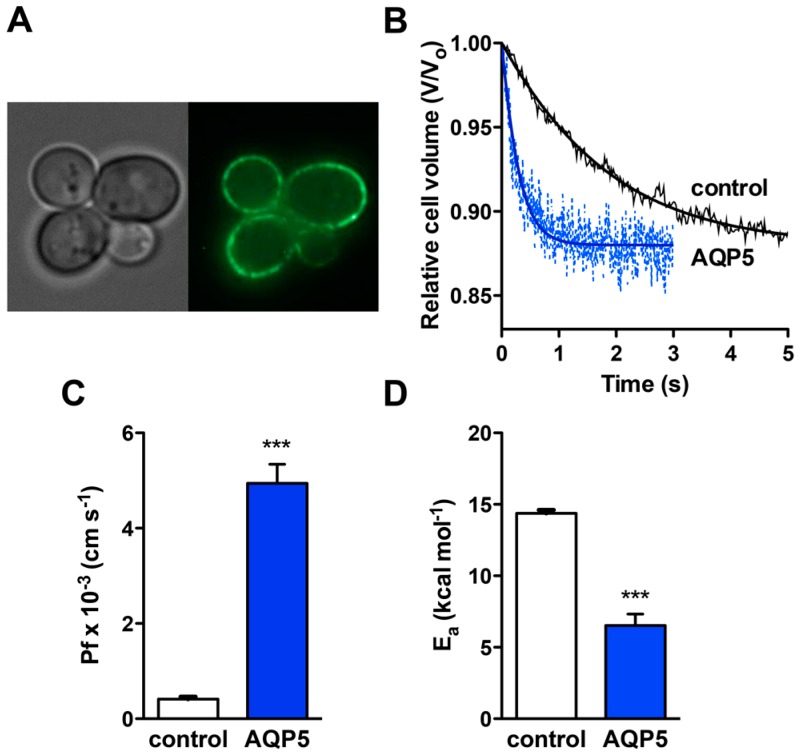
Expression and function of rat AQP5 (Aquaporin-5) in yeast. (**A**) Epifluorescence images of GFP-tagged AQP5 localization (green) in yeast cells (100× objective); (**B**) Representative time course of the relative cell volume (*V*/*V*_0_) changes after a hyperosmotic shock inducing cell shrinkage (pH 7.4); (**C**) Water permeability coefficients of control (Pf = (0.41 ± 0.05) × 10^−3^ cm·s^−1^) and cells expressing AQP5 (Pf = (4.94 ± 0.40) × 10^−3^ cm·s^−1^), measured at 23 °C and pH 7.4. Data are mean ± SD of 10 measurements; (**D**) Activation energies (E_a_) for water permeation of control and AQP5 cells (15.16 ± 0.85 and 6.52 ± 0.82 kcal·mol^−1^, respectively). Data are mean ± SD. *** *p* < 0.001.

Yeast cells made devoid of endogenous aquaporins (aqy-null) were transformed with either the empty plasmid pUG35 (control cells) or the plasmid containing the rat AQP5 gene (mentioned as AQP5 cells, for clarity). The expression of AQP5 in the *S. cerevisiae* model was assessed by fluorescence microscopy, using GFP tagging. In transformed cells, AQP5–GFP is localized at the cellular membrane, as depicted in Figure 1A.

The permeability of yeast expressing AQP5 was evaluated by stopped-flow fluorescence after loading cells with the volume sensitive dye carboxifluorescein. When cells are exposed to hyperosmotic shock with impermeant solutes, water outflow induces cell shrinkage. Water permeability is then evaluated by monitoring the time course of fluorescence output that reflects the transient volume change.

As depicted in Figure 1B, cells expressing AQP5 show a much faster volume change after a hyperosmotic shock. The water permeability coefficient Pf was 12-fold higher for AQP5 cells ((4.94 ± 0.40) × 10^−3^ cm·s^−1^ and (0.41 ± 0.05) × 10^−3^ cm·s^−1^ for AQP5 and control, respectively) (Figure 1C). The activation energy for water transport E_a_ was concomitantly lower for AQP5 cells (6.52 ± 0.82 kcal·mol^−1^) compared to the control (15.16 ± 0.85 kcal·mol^−1^) (Figure 1D), corroborating the increase in membrane water permeability conferred by AQP5 expression. Although AQP5 behavior as a water channel is well known in the literature, these data validate the use of the yeast system to detect AQP5 function and further explore mechanisms of regulation.

### 2.2. Effect of pH and Glucose-Stimulated Phosphorylation on Rat AQP5 Permeability

AQPs can be subjected to regulation via different mechanisms, among which pH regulation has been disclosed for plant [28] and for a few mammalian AQPs, such as AQP0, AQP3, and AQP6 [30,31,32]. It is also known that eukaryotic AQPs can be gated by phosphorylation [24].

A gating mechanism regulating human AQP5 activity has been proposed by molecular dynamics simulations [33]. This study revealed that the AQP5 channel could change between an open and closed state by a tap-like mechanism at the cytoplasmic end, induced by a translation of the His67 inside the pore, blocking the entrance of the channel. Moreover, when in the open state, the selectivity filter (SF) can regulate the flow rate of water molecules by exhibiting two different conformations (wide or narrow). These two conformations are decided by the side chain orientation of His173 and the proximity to Ser183—when His173 is close to Ser183, the SF is in the narrow conformation and the water passage is restricted. The trigger for this gating mechanism has not been described; in addition, this in silico approach has not been so far experimentally validated. A similar gating mechanism for human AQP4 was recently described [34], where two putative gate regions formed by two residues on the cytoplasmic side (His95 and Cys178) and the other two on the SF region (Arg216 and His201) modulate opening and closure of the AQP4 pore along four possible conformational states. The relative stability of the two resulting states, open and closed, may depend on small changes in the microenvironment, such as variations of pH. Indeed, a pH-dependent gating mechanism was recently obtained from in silico and in vitro studies [35], ascribing to His95 located in AQP4 cytoplasmic end the role of regulating channel permeability. Phosphorylation of AQP4 has also been demonstrated with opposed effects depending on the residue that is phosphorylated. AQP4 is inhibited when Ser180 is phosphorylated in loop D and is activated when Ser111 in loop B is phosphorylated [36]. These observations prompted us to investigate if AQP5 would be gated by pH or by phosphorylation.

Our group has recently characterized the pH dependence of AQP3 permeability using the yeast heterologous expression system [27]. Thus, we first decided to investigate the effect of external pH on AQP5 activity of rat AQP5-transformed yeasts. Although our yeast cells express rat AQP5, sequence alignment of human and rat AQP5 isoforms showed a sequence identity of 91% [37] (Figure S1). From the analyses of the amino acid sequences, we can infer that human and rat AQP5 may share the same gating mechanism.

**Figure 2:**
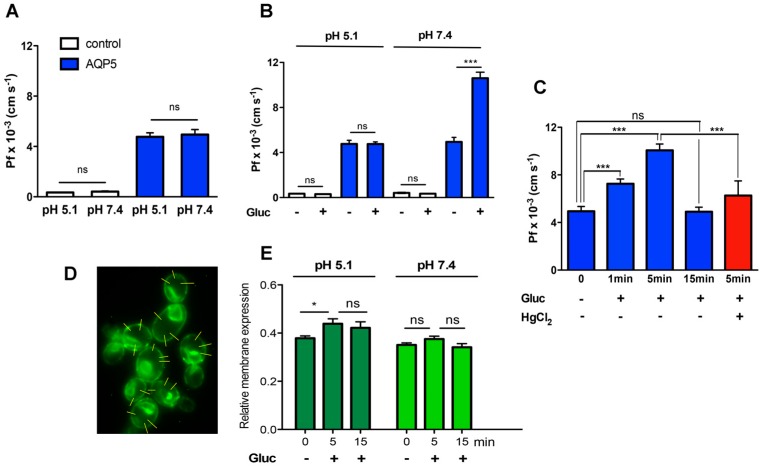
Regulation of AQP5 water permeability. (**A**) Water permeability Pf at pH 5.1 and pH 7.4 of control cells (Pf = (0.35 ± 0.01) × 10^−3^ and (0.41 ± 0.05) × 10^−3^ cm·s^−1^, respectively) and yeast cells expressing AQP5 (Pf = (4.77 ± 0.32) × 10^−3^ and (4.94 ± 0.40) × 10^−3^ cm·s^−1^, respectively); (**B**) Water permeability Pf at pH 5.1 and pH 7.4 upon an external glucose pulse (**C**) Time course of glucose-induced phosphorylation (1, 5 and 15 min, pH 7.4) and inhibition of AQP5 by HgCl_2_ 0.05 mM. Data are mean ± SD of 10 measurements; (**D**) Representative epifluorescence images of GFP-tagged AQP5 localization in yeast cells (100× objective); linear intensity profiles are indicated (**yellow lines**); and (**E**) Relative membrane expression of AQP5 calculated from fluorescence intensity profiles (30 cells in each experimental condition, 3 profiles for each cell, from at 3 independent experiments). ns, non significant, * *p* < 0.5, *** *p* < 0.001.

Since the physiological pH of yeast’s natural environment is acidic (3.5–6.5) [38], and conditions at which cells show optimal growth and expression and trafficking mechanisms are expected to be fully active, we chose the external pH 5.1 to test yeast membrane water permeability. In addition, considering that a mammalian aquaporin is being expressed, permeability was also measured at the mammalian physiological pH 7.4. Permeability experiments with AQP5 and control cells incubated at pH 5.1 and 7.4 showed that, by changing external pH channel, activity was not altered (Figure 2A). At pH 5.1, Pf = (0.35 ± 0.01) × 10^−3^ cm·s^−1^ and E_a_ = 14.38 ± 0.22 kcal·mol^−1^ for control cells, and Pf = (4.77 ± 0.32) × 10^−3^ cm·s^−1^ and E_a_ = 7.69 ± 0.86 kcal·mol^−1^ for AQP5 cells, were not different from the respective values at pH 7.4 (detailed above, Figure 1). These results indicate that the acidic external pH does not affect AQP5 water permeability.

It is known that AQP5 expression and trafficking can be regulated by phosphorylation, but whether phosphorylation also regulates channel activity and contributes to gating still remains uncertain. Several studies reported AQP5 redistribution in plasma membrane of animal cells initiated by phosphorylation [20,39]. Post-transcriptional regulation of AQP5 function in response to stimuli such as neurotransmitters, hormones, and cyclic adenosine monophosphate (cAMP), has been reported (for a review see [7]). cAMP regulates aquaporin-5 expression at both transcriptional and post-transcriptional levels through a protein kinase A (PKA) pathway [40], increasing AQP5 abundance on the apical membrane of lung epithelial cells after long-term exposure [41]. Besides translocation to the plasma membrane, phosphorylation of AQP5 was shown to promote cell proliferation [8] and, interestingly, AQP5 Ser156 was found preferentially phosphorylated in tumor cells [42], supporting AQP5 phosphorylation involvement in cell proliferation.

In yeast *S. cerevisiae*, the basal intracellular cAMP concentration is low [43]. However, addition of glucose or related fermentable sugars after a period of glucose-starvation triggers the Ras/PKA pathway, creating a sudden and transient increase in intracellular cAMP levels that induce a protein phosphorylation cascade [44,45]. Activation of this pathway by glucose mimics the well-known hormonal-induced phosphorylation pathways that occur in animal cells [46]. Therefore, to examine the effect of phosphorylation on AQP5 water permeability, glucose starved yeast cells were incubated with 100 mM glucose for 5 min before Pf measurements. As shown in Figure 2B, glucose addition did not affect Pf of control or AQP5 cells at pH 5.1 (Figure 2B). However, at pH 7.4, glucose pulse resulted in a significantly two-fold increased Pf in AQP5 cells (Pf = (10.60 ± 0.53) × 10^−3^ cm·s^−1^) and a 30-fold increase compared with basal levels of control cells (Figure 2B).

In addition, we investigated the time course of AQP5 activation after glucose addition at pH 7.4 (Figure 2C). Following a glucose pulse, a transient strong increase of cAMP with peak values around 1–2 min that progressively decay to about their basal levels, was previously reported in yeast cells [47,48]. Our data show a significant increase of AQP5 water permeability after 1 min that was further increased at 5 min; after 15 min, glucose exposure no longer produces effect on AQP5 permeability, possibly due to the decay of cAMP synthesis. Yeast cells were subsequently incubated for 5 min simultaneously with glucose and HgCl_2_, a well-known aquaporin inhibitor (Figure 2C). In this case, the glucose-induced increase in Pf of AQP5 cells was partially abolished (Pf = (6.26 ± 1.23) × 10^−3^ cm·s^−1^. The inhibitor alone had no effect on Pf of control cells.

Afterwards, to determine whether cAMP-mediated increase in water permeability was due to AQP5 increased trafficking and abundance or to opening of the channel, we measured GFP-tagged AQP5 relative membrane expression (Figure 2D). Before glucose addition, the membrane abundance measured at pH 5.1 and pH 7.4 was not significantly different (0.37 ± 0.07 and 0.35 ± 0.07, respectively) (Figure 2E). Although membrane abundance was slightly increased after 5 min glucose pulse (implying that also trafficking is triggered by phosphorylation, as previously reported by Kitchen et al. [20]), this effect was only detected at extracellular pH 5.1 (0.49 ± 0.08 and 0.47 ± 0.12 at 5 and 15 min, respectively). Interestingly, at pH 7.4, no significant difference could be detected after 5 min (0.37 ± 0.07), and at both pHs the membrane abundance was kept stable at least for 15 min (Figure 2E).

Also to consider is the fact that, if a carbon source is available, external shifts in proton concentration in the pH range from 3.0 to 7.5 do not significantly affect yeast internal pH values due to ATPase activity [38]. However, a two-fold increase in Pf with concomitant reduction of E_a_ after glucose addition only happened at pH 7.4 (Figure 2B). No increase was seen at pH 5.1. Interestingly, pH 5.1 is in the range of yeast physiological pH at which the machineries for protein expression, transcription, and trafficking are expected to be in place. If the increase in Pf observed would be simply due to an increase in AQP5 membrane abundance, then it should also be detected at pH 5.1. Hence, since we observe differences in permeability mediated by phosphorylation when the external pH is changed, changes in the channel structure activity rather than in AQP5 membrane abundance might be responsible for the measured difference in permeability.

**Figure 3:**
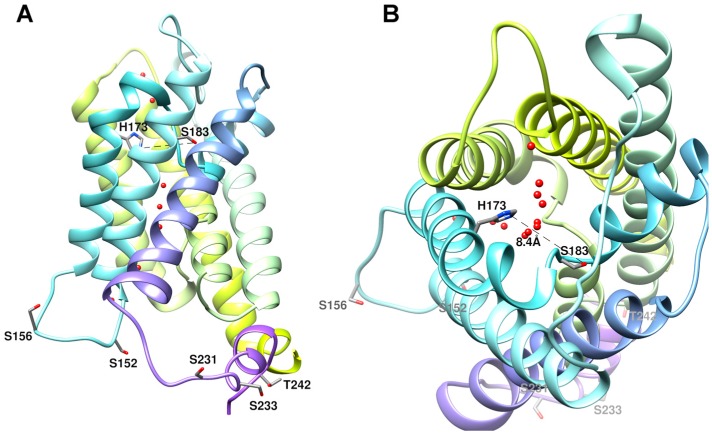
Structure of human AQP5 monomer. (**A**) Side view of the monomer with several phosphorylation consensus sites in the cytoplasmic region (Ser152, Ser156, Ser231, Ser233, and Thr242) shown in licorice representation. Thr259, also a phosphorylation site, is not represented due to the inexistent electron density beyond Pro245 for hAQP5 structure [49]; (**B**) Top view of the monomer with His173 and Ser183 in the selectivity filter (SF) shown in licorice representation. The distance between these two residues (8.4 Å) corresponds to the proposed distance for the SF wide conformation [33]. Water molecules are shown as red spheres along the channel pore. Structures were generated with Chimera (http://www.cgl.ucsf.edu/chimera) and are based on AQP5 X-ray structure (PDB databank code 3D9S).

Figure 3A displays the structure of human AQP5 with several consensus phosphorylation sites at cytoplasmic loop D (Ser152 and Ser156) and at C-terminal (Ser231, Ser233, and Thr242) [49]. In Figure 3B the top view of the monomer is depicted, with His173 and Ser183 located in the selectivity filter (SF). The distance between these two residues (8.4 Å) was proposed to correspond to the pore wide conformation [33]. All these residues are conserved in rat AQP5 sequence (Figure S1) with the exception of Ser233. Phosphorylation of Ser156 was reported to play an important role in AQP5 translocation to the plasma membrane in HEK293 cells [20]. In contrast, another study observed that a mutation on Ser156 had no effect on membrane trafficking but instead affected cell proliferation [42].

Thr259 is not represented in Figure 3A due to the inexistent electron density beyond Pro245 in hAQP5 structure. However, this residue is also an interesting phosphorylation site due to the homology to Ser256 in hAQP2. It has been demonstrated that phosphorylation of Ser256, besides triggering AQP2 insertion at apical plasma membrane, is also essential to modulate AQP2 function, increasing water permeability of the individual channel [50,51]. In a recent study, extracellular acidic pH was shown to attenuate AQP2 hormone-induced phosphorylation and membrane apical trafficking, probably by inhibition of vasopressin V2 receptor-G protein-cAMP-PKA actions [52].

From a structural point of view, Ser152 and Ser156 are strong candidates prone to induce conformation changes at loop D with impact on the protein channel monomeric conformation. However, phosphomimetic mutations of Ser156 were able to increase membrane expression but did not cause any significant structural change [20]. It is thus reasonable to anticipate that more than one phosphorylation site is necessary to produce a measurable conformation change. Phosphorylated AQP5 (Ser and Thr residues facing the cytoplasmic region, Figure 3A) may induce a change in channel conformation, and in this new conformation de-protonation of His183 residue (facing the outer membrane) may occur at pH 7.4, with widening of the channel pore (Figure 3B). While at pH 7.4 the channel is wide open, at pH 5.1 the protonated residues and putative hydrogen-bond interactions hold the channel in the narrow open conformation, with lower permeability. Interestingly, the human AQP5 crystals were obtained at pH 7.0–7.6, the pH range where, in this study, phosphorylated AQP5 shows increased permeability.

### 2.3. Hydrogen Peroxide Consumption

AQP5 has been implicated in carcinogenesis and its tissue expression might be associated with cancer aggressiveness [7]. The mechanisms underlying AQP5 involvement in cancer are still unclear, but in addition to participation in intracellular signaling transduction pathways and interaction with oncoproteins, AQP5 channel activity facilitating rapid changes in cell volume and subsequent changes in cell shape, was proposed to be crucial for cell migration [7,11]. Furthermore, AQP5 permeation of H_2_O_2_ and subsequent implication in cell oxidative stress would help explaining its participation in tumorigenesis.

**Figure 4:**
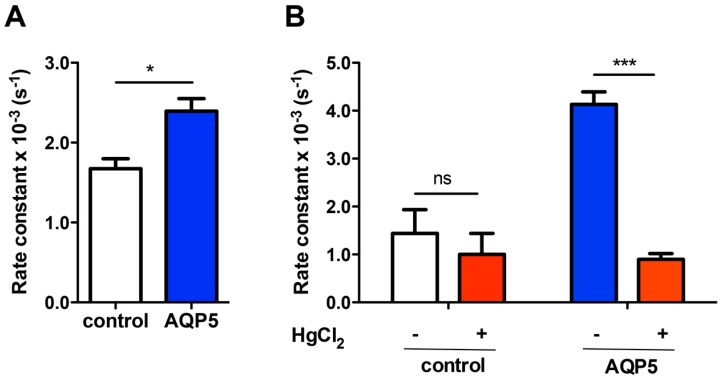
AQP5-dependent H_2_O_2_ consumption of yeast cells. (**A**) First-order kinetic rate constant (s^−1^) of H_2_O_2_ consumption measured with the Clark electrode (O_2_ measurement); (**B**) First-order kinetic rate constant (s^−1^) of the H_2_O_2_ consumption measured with the H_2_O_2_ electrode, before (**white and blue bars**) and after incubation with 0.5mM HgCl_2_, 5min at RT (**red bars**). Values are mean ± SD of triplicates. ns, non significant, * *p* < 0.05, *** *p* < 0.001.

To investigate if AQP5 expression increases the rate of H_2_O_2_ diffusion through membranes, we measured the consumption of external H_2_O_2_ in control and AQP5 expressing cells by electrochemical assays, using O_2_- and H_2_O_2_-specific electrodes. After the external addition of H_2_O_2_, the rate constant of O_2_ consumption by cells was 1.4-fold increased (*p* < 0.05) in AQP5 cells (k_Control_ = (1.68 ± 0.12) × 10^−3^ s^−1^) compared to control (k_AQP5_ = (2.39 ± 0.15) × 10^−3^ s^−1^) (Figure 4A), indicating that AQP5 cell membranes possess a facilitated H_2_O_2_ diffusion pathway. To validate this result and further investigate if AQP5 would be mediating H_2_O_2_ permeation, we then followed H_2_O_2_ cell consumption using a specific H_2_O_2_ electrode and checked whether the aquaporin inhibitor HgCl_2_ quenches the uptake. The obtained results (k_Control_ = (1.44 ± 0.49) × 10^−3^ s^−1^ and k_AQP5_ = (4.13 ± 0.26) × 10^−3^ s^−1^) corroborate the previous increased diffusion rate of H_2_O_2_ consumption by AQP5 cells (Figure 4B). In addition, HgCl_2_ showed a significant inhibitory effect, reducing aproximately five-fold the rate of consumprion (*p* < 0.001) and not affecting the control. Therefore, these data strongly suggest that AQP5 can mediate H_2_O_2_ diffusion through membranes.

### 2.4. AQP5 Implication on Cell Oxidative Status

**Figure 5:**
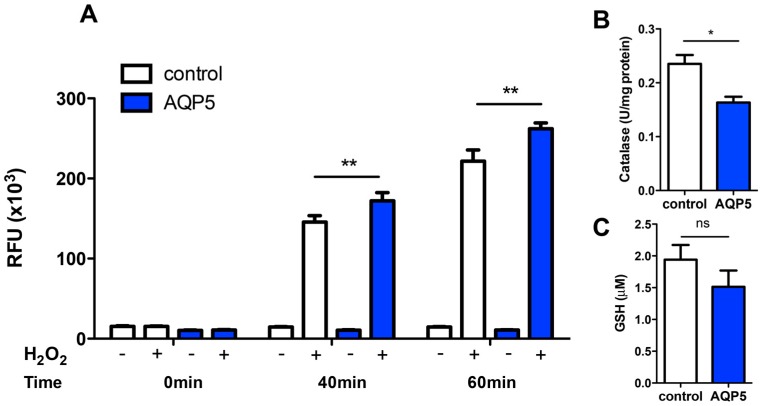
Cellular levels of ROS (oxidant), GSH and catalase (antioxidants). (**A**) Time course of Intracellular ROS production after acute stress induction with 20 mM H_2_O_2_; (**B**) Catalase activity and (**C**) total intracellular GSH content of yeast strains. Values are means ± SD of triplicates, ns, non significant, * *p* < 0.05, ** *p* < 0.01.

In order to assure that the disappearance of extracellular H_2_O_2_ was due to cellular uptake rather than extracellular degradation, we measured the intracellular levels of ROS after acute stress induction with 20 mM H_2_O_2_. As expected, a higher intracellular level of ROS was detected for AQP5 cells (Figure 5A). Although control cells also respond to oxidative stress induction, which may be explained by basal H_2_O_2_ membrane lipid diffusion [53], ROS content was significantly increased in AQP5 cells after approximately 40 min of stress induction. Thus, the extracellular disappearance of H_2_O_2_ measured by electrodes is in agreement with intracellular ROS production, supporting AQP5-dependent H_2_O_2_ consumption.

To further confirm that the increase in external H_2_O_2_ consumption and concomitant intracellular ROS levels were mainly due to AQP5 expression and activity, we assessed the cells’ antioxidant defense system by measuring catalase activity and GSH level in basal conditions (before addition of H_2_O_2_). An increase in these scavengers could explain the previously obtained results without the influence of AQP5.

Catalase activity, as part of the antioxidative defense system, was 30% reduced (0.235 ± 0.012 and 0.163 ± 0.008 U/mg protein, for control and AQP5, respectively) (Figure 4B) and GSH level was also slightly diminished in AQP5 cells (1.94 ± 0.18 µM for control and 1.53 ± 0.20 µM for AQP5) (Figure 5C), indicating that these scavengers are not contributing for the observed higher H_2_O_2_ consumption and ROS production in AQP5 cells.

### 2.5. Yeast Sensitivity to Hydrogen Peroxide

ROS are no longer known only as purely harmful but instead they are described as important regulators of signaling pathways [54]. The increased intracellular levels of ROS in AQP5 cells after acute stress induction with H_2_O_2_ prompted us to investigate its outcome on yeast cell survival at shorter and longer periods after inducing the oxidative stress.

**Figure 6:**
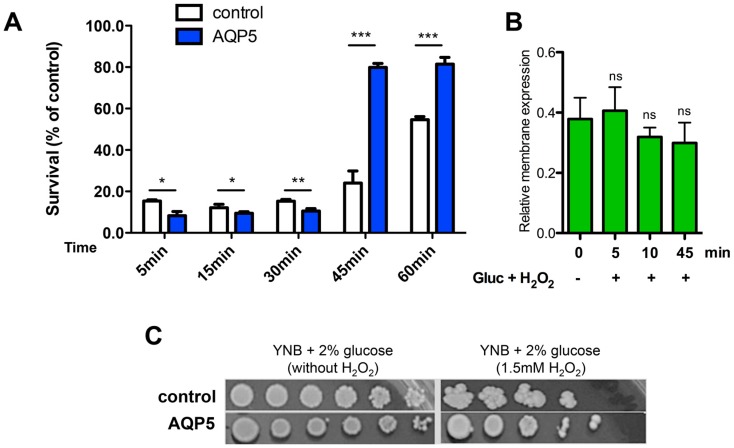
Stress response of yeast strains to H_2_O_2_. (**A**) Acute stress response. Time course of cell survival of yeast cells in liquid culture after treatment with 0.5 mM H_2_O_2_. Percentage survival is expressed relative to untreated controls. Values are means ± SD of triplicates. * *p* < 0.05, ** *p* < 0.01, *** *p* < 0.001; (**B**) Relative membrane expression of AQP5 calculated from fluorescence intensity profiles (30 cells in each experimental condition, 3 profiles for each cell, from 3 independent experiments). ns, non significant; (**C**) Chronic stress response. Growth assay of yeast cells under oxidative stress. Yeast suspensions were spotted in 10-fold dilution on solid YNB plates without or with 1.5 mM H_2_O_2_. Growth was recorded after two weeks at 28 °C. Photographs shown are representative of three independent experiments with consistent results.

In a first approach, cells suspended in liquid medium were treated with 0.5 mM H_2_O_2_ and, after incubation for different time intervals, the ability of plated cells to survive and form colonies was evaluated. Interestingly, as depicted in Figure 6A, a different and opposed behavior was detected in a 60 min time range. While initially AQP5 cells were more sensitive to H_2_O_2_ than control, longer incubation periods, for more than 30 min, reveal that AQP5 cells survive better and are significantly more resistant to the oxidative stress insult. Thus, the initial H_2_O_2_-triggered harmful effects and lower cell survival for AQP5 cells (*p* < 0.05 and *p* < 0.01) are reversed after a while, showing that after 45 min incubation cells are able to survive significantly better than control (*p* < 0.001). It is worth mentioning that the initial sensitivity of AQP5 cells may be due to the cells’ lower antioxidant defense system, as suggested by catalase activity and GSH level, thus rendering AQP5 cells more prone to ROS effects. However, after 45 min of incubation with H_2_O_2_, it is clear that AQP5 cells are more resistant than control.

Measurements of membrane abundance showed that AQP5 expression was not affected after treatment with 0.5 mM H_2_O_2_ (Figure 6B). Indeed, these results are expected from the cloning method as the AQP5 gene was cloned under inducible MET25 promoter. MET25 promoter provides an inducible expression system in the absence of methionine, but renders the expression of AQP5 insensitive to stimuli or inhibition, which would normally occur, such as possible regulation by oxidative challenge.

These same conclusions can be drawn when cells were grown in solid media containing H_2_O_2_ (Figure 6C). In this assay, the long-term stress response given by the ability to grow under oxidative stress was evaluated after two weeks. A clear resistance of AQP5 cells can be observed.

Altogether, these results indicate that AQP5 expression contributes to activation of either the cell antioxidant defense response or the signaling transduction pathways triggered by H_2_O_2_. The increased resistance displayed by AQP5 cells might be the result of a faster metabolic adaptation to the oxidative cell status conferred by AQP5 expression.

## 3. Discussion

The present study provides experimental evidences for the direct regulation of AQP5 water permeability by pH dependent on phosphorylation and for AQP5 involvement in cell oxidative stress response.

Human AQP5 was the first aquaporin crystalized in full tetrameric assembly [49] and its trafficking and membrane expression are regulated by phosphorylation [20] through PKA and RAS signaling pathways that promote cell proliferation [8]. The fact that AQP5 was found preferentially phosphorylated in tumor cells strongly suggests that its regulation might be involved in tumorigenesis. In addition to trafficking, AQP5 channel activity may be crucial for cancer cell migration and proliferation [7,11]. Therefore, it is reasonable to hypothesize that AQP5 ability to permeate H_2_O_2_ in addition to water, could also contribute to its up-regulation in cancer tissues.

In this context, this study aimed at investigating two mechanisms supporting AQP5 involvement in tumorigenesis: (i) its ability to be short-term regulated or gated, either by acidic conditions or by phosphorylation; and (ii) its ability to permeate H_2_O_2_ in addition to water and consequent outcome in oxidative cell response.

Our data show that AQP5 can be gated by pH in a phosphorylation dependent manner. Glucose addition with consequent PKA pathway activation may simultaneously stimulate AQP5 trafficking and abundance at the plasma membrane and contribute to opening of the channel at physiological pH 7.4; the measured increase in water permeability in AQP5 cells may reflect the sum of the two processes, trafficking and gating.

In our assays, a small increase in AQP5 membrane expression 5 min after the glucose pulse was detected, but only for extracellular pH 5.1. At pH 7.4 the increase in membrane abundance was not observed and cannot explain the higher Pf measured. It is worth mentioning that, although at different extracellular pHs, yeasts are able to maintain the intracellular pH close to neutrality if a carbon source is available. Thus, the extracellular pH is not expected to greatly influence inner pH and is not likely to induce changes in membrane trafficking. In fact, the average membrane expression was similar at both pHs. Since phosphorylation occurs intracellularly, one may speculate that it is the direct AQP5-phosphorylation that alters protein conformation and, in this new conformation, channel widening results from de-protonation of residues at pH 7.4. Therefore, in addition to promoting membrane trafficking, phosphorylation may endorse an AQP5 channel with the ability of pH sensing.

At mammalian physiologic conditions (pH 7.4) phosphorylated AQP5 enables larger fluxes of water through membranes, which may contribute to rapid changes in cell volume and shape and thus facilitate cell migration. Extracellular pH shifts acidic conditions favors channel narrowing, allowing fine-tuning of cell volume. Notably, although natural for yeast cells, the low pH tested is far below the pH found in mammalian tissues, even in solid tumors where it can reach pH 6.5 [55]. There are, however, a few mammalian acidic physiological conditions where pH gets close to this value. For instance, sweat shows acidic pH levels, between 4.6 and 5.4 [56], and in the skin the protective sweat acid mantle acidity ranges from 4 to 5.5 [56]; the pH of human stomach is highly acidic, usually from 1 to 2; osteoclasts’ acidic microenvironment below pH 5.5 is critical for the bone resorption [57]; an acidic pH luminal fluid microenvironment is important for sperm maturation [58]. Interestingly, AQP5 was found expressed in sweat glands [59], gastric mucosa [60], bone cells [61], and in the epididymis [58]. In all these tissues, effective mechanisms of water flux regulation induced by pH fluctuations might be advantageous to prevent excessive water reabsorption and control cell volume and shape.

Furthermore, this study demonstrates that AQP5 is able to permeate H_2_O_2_. AQP5-transformed cells show a high rate of H_2_O_2_ consumption, indicating that AQP5 facilitates H_2_O_2_ membrane permeation. Data of H_2_O_2_ consumption, ROS intracellular levels, and resistance to H_2_O_2_ suggest that AQP5 is involved in cell oxidative stress response and point to a novel mechanism explaining AQP5 contribution to tumorigenesis.

Noteworthy, the initial higher sensitivity to H_2_O_2_ displayed by AQP5-expressing cells turned into higher resistance in chronic oxidative stress conditions. Yeast cells possess a limited pool of antioxidant enzymes that protect against ROS but are not sufficient to protect cells from sudden and high oxidative challenges. The onset of oxidative stress usually induces an early response, where the intracellular antioxidant system provides instant protection against the initial toxic accumulation of ROS. Stress signals such as H_2_O_2_ can activate transcription factors which upregulate the expression of many genes including the ones encoding enzymatic (e.g., catalases) and non-enzymatic antioxidants (e.g., GSH) [62]. The subsequent syntheses of antioxidant defenses promote ROS scavenging, repair oxidized biomolecules, and restore cellular redox balance [63,64]. These response mechanisms help ensure the survival of non-lethally damaged cells [54]. Additionally, exposure of cells to severe oxidative stress can elicit lethal response pathways such as autophagy, apoptosis, and necrosis [64]. We may speculate that AQP5-facilitated H_2_O_2_ permeation induces a faster activation of transcription factors and antioxidant system stimulation, contributing to the pro-survival cellular responses to oxidative stress.

The results obtained in this study with yeast cells can possibly be translated to mammalian cells. For instance, *S. cerevisiae* contains three conserved signaling modules that control transcriptional regulation triggered by oxidative stress, and two of them have homology to others species, including mammalian [65]. Furthermore, the activating protein-1 (AP-1) family of transcription factors that regulate cellular processes such as proliferation, differentiation, apoptosis, and stress response, exists in both mammalian and yeast cells and can be activated by H_2_O_2_ [66]. In yeast, the AP-1 homolog Yap1 functions as an oxidative stress sensor and regulates the expression of antioxidant genes in response to stress, including thioredoxin, thioredoxin reductase, glutathione reductase, and γ-glutamylcysteine synthase [65], and, interestingly, the AQP5 5’-flanking region has a consensus binding site for AP-1 [67]. Thus, a mechanism where H_2_O_2_ permeated by AQP5 contributes to activation of AP-1 transcription factor that in turn stimulates antioxidant genes expression and possibly AQP5 gene expression as well, cannot be disregarded. A major issue that remains to be resolved is the precise connection between AQP5 phosphorylation via the cAMP-PKA pathway and the oxidative stress cell resistance conferred by AQP5. Further experiments using mammalian cells and the human AQP5 gene will help to untangle the mechanisms underlying AQP5 involvement in oxidative stress response.

## 4. Materials and Methods

### 4.1. Yeast Strains and Growth Conditions

Plasmid (pcDNA3) with *Rattus norvegicus* AQP5 cDNA (pcDNA3-AQP5), kindly provided by Prof. Miriam Eschevarria, Virgen del Rocio University Hospital, Seville, Spain, was used for AQP5 cDNA amplification. The centromeric plasmid pUG35 was used for cloning AQP5, conferring C-terminal GFP tagging, MET25 promoter, and CYC1-T terminator [68].

For plasmids propagation, *Escherichia coli* DH5α was used as host [69]. *E. coli* transformants were maintained and grown in Luria-Bertani broth (LB) supplemented with ampicillin (100 µg·mL^−1^), at 37 °C [70]. Plasmid DNA was extracted from *E. coli* using a GenElute^TM^ Plasmid Miniprep Kit (Sigma-Aldrich, St. Louis, MO, USA).

*Saccharomyces cerevisiae* (10560-6B MATa leu2::hisG tpr1::hisG his3::hisG ura3-52 aqy1D::KanMX aqy2D::KanMX) from now on designated as aqy-null, was used as host strain for heterologous expression of AQP5. The aqy-null strain was grown and maintained in YPD medium (2% *w*/*v* peptone, 1% *w*/*v* yeast extract, 2% *w*/*v* glucose). Transformed yeast strain was grown in YNB medium (2% *w*/*v* glucose, 0.67% (DIFCO) Yeast Nitrogen Base) supplemented with the adequate requirements for prototrophic growth [71] and maintained in the same medium with 2% (*w*/*v*) agar. For stopped-flow assays, the same medium was used for yeast cell growth. For all experiments, cells were grown to mid exponential phase (OD_600_ 1.0).

### 4.2. Cloning and Heterologous Expression of AQP5 in S. cerevisiae

This section was performed according to previously described methods [27]. Briefly, after propagation, isolation, and purification of pcDNA3_AQP5, AQP5-specific primers modified to incorporate restriction sites for *SpeI* (underlined) and *ClaI* (underlined) (5′-GGACTAGTCCT ATG AAA AAG GAG GTG TGC TCC CTT GC-3′ and 5′-CCATCGATGGA GTG TGC CGT CAG CTC GAT G-3′, respectively) were designed and used for PCR amplification of AQP5 cDNA (carried out in an Eppendorff thermocycler using Taq Change DNA polymerase from NZYTech, Lisbon, Portugal). The PCR product was digested with *SpeI* and *ClaI* restriction enzymes, purified (using a Wizard^®^ SV Gel and the PCR Clean-Up System kit Promega) and cloned (using T4 DNA Ligase Roche) into the corresponding restriction sites of pUG35 digested with the same restriction enzymes, behind the MET25 promoter and in frame with the GFP sequence and CYC1-T terminator, according to standard protocols [70], to construct the expression plasmid pUG35-AQP5.

This plasmid was propagated in *E. coli* DH5α. After extraction and purification, fidelity of constructs and correct orientation of AQP5-cDNA were verified by PCR amplification and DNA sequencing. Transformation of the *S. cerevisiae* aqy-null strain with pUG35-AQP5 was performed using the lithium acetate method described in [72], from now on named AQP5 strain, for clarity. The same strain was also transformed using an empty pUG35 vector (which does not contain AQP5 cDNA) to be used as a control (further indicated as control strain). Transformants were selected on YNB medium without uracil as auxotrofic marker.

### 4.3. AQP5 Subcellular Location by Fluorescence Microscopy

For subcellular localization of GFP-tagged AQP5 in *S. cerevisiae*, yeast cells in the mid-exponential phase were observed using a Zeiss Axiovert 200 fluorescence microscope (Zeiss, Jena, Germany), at 495 nm excitation and 535 nm emission wavelengths. Fluorescence microscopy images were captured with a digital camera (CoolSNAP EZ, Photometrics, Tucson, AZ, USA) and using the Metafluor software (Molecular Devices, Sunyvale, CA, USA).

AQP5 membrane expression was measured by evaluating GFP-protein fluorescence intensity according to [20,73]. A linear profile that crosses the cell membrane was generated and analyzed using the software ImageJ (https://imagej.net). The intensity profile along the line path from at least 30 cells in each experimental condition (*n* = 3) was recorded (Figure 2D), and for each cell three profile lines were taken. The background intensity along the same distance was measured and subtracted from the peak fluorescence intensity over each line, and the obtained difference divided by the maximal fluorescence to calculate the relative membrane expression.

### 4.4. Cell Sampling and CFDA Loading

For water permeability assays, yeast transformants grown up to OD_600_ 1, were harvested by centrifugation (5000× *g*; 5 min; 4 °C) (Allegra^®^ 6 Series Centrifuges, Beckman Coulter^®^, Brea, CA, USA), washed 3 times and resuspended in ice cold sorbitol (1.4 M) K^+^-citrate (50 mM pH 5.1 or 7.8) buffer up to a concentration of 0.3 g·mL^−1^ wet weight and cells were incubated on ice for at least 90 min. Prior to permeability assays, cells were preloaded with the non-fluorescent precursor 5(6)-carboxyfluorescein diacetate (CFDA, 1 mM, 10 min at 30 °C), which is intracellularly hydrolyzed yielding the impermeable fluorescent form (CF). Cells were then diluted (1:10) in 1.4 M sorbitol buffer and immediately used for experiments.

### 4.5. Cell Volume Measurements

Equilibrium cell volumes (*V*_o_) were obtained after loading the cells with CFDA under an epifluorescent microscope (Zeiss Axiovert, Zeiss, Jena, Germany) equipped with a digital camera. Cells were assumed to have a spherical shape with a diameter calculated as the average of the maximum and minimum dimensions of each cell.

### 4.6. Water Permeability Assays

Permeability assays were performed by stopped-flow fluorescence spectroscopy as previously described [74], using a HI-TECH Scientific PQ/SF-53 stopped-flow apparatus, which has a 2-ms dead time, controlled temperature, interfaced with a microcomputer.

Experiments were performed at temperatures ranging from 9 to 34 °C. Four runs were usually stored and analyzed in each experimental condition. In each run, 0.1 mL of cell suspension was mixed with an equal volume of hyperosmotic sorbitol buffer (2.1 M sorbitol, 50 mM K-citrate, pH 5.1 or 7.4) producing an inwardly directed gradient of the impermeant sorbitol solute that induces water outflow and cell shrinkage. Fluorescence was excited using a 470 nm interference filter and detected using a 530 nm cut-off filter. The time course of cell volume change was followed by fluorescence quenching of the entrapped fluorophore (CF). The recorded fluorescence signals were fitted to a single exponential from which the rate constant (*k*) was calculated.

The osmotic water permeability coefficient, Pf, was estimated from the linear relationship between Pf and *k* [74], Pf = *k*(*V*_o_/*A*)(1/*V*_w_(*osm*_out_)), where *V*_w_ is the molar volume of water, *V*_o_/*A* is the initial volume to area ratio of the cell population, and (*osm*_out_) is the final medium osmolarity after the osmotic shock. The osmolarity of each solution was determined from freezing point depression by a semi-micro-osmometer (Knauer GmbH, Berlin, Germany). The activation energy (E_a_) of water transport was evaluated from the slope of the Arrhenius plot (ln Pf as a function of 1/T) multiplied by the gas constant *R*.

### 4.7. External pH Dependence and In Vivo PKA Phosphorylation

Yeast cells were grown and prepared as above-described and incubated in isotonic sorbitol buffer (1.4 M sorbitol, 50 mM K-citrate) at two different pH (5.1 and 7.4) for at least 90 min. Deprived of a carbon source and incubated in ice for a long period, yeast cells are considered in starvation. The production of intracellular cAMP and phosphorylation was triggered immediately before water permeability measurements by the addition of 0.1 M glucose (adjusted to pH 5.1 or 7.4) to starved cells [44,45].

### 4.8. Hydrogen Peroxide Consumption

The consumption of H_2_O_2_ was measured in intact cells. Cells were harvested by centrifugation (5000× *g*; 10 min at RT) (Hettich Rotofix32, Tuttlingen, Germany), resuspended in fresh growth media and incubated at 30 °C with orbital shaking. Hydrogen peroxide (50 µM) was added to intact cells and the consumption of H_2_O_2_ was measured by following O_2_ release with an oxygen electrode (Hansatech Instruments Ltd., Norfolk, UK) after the addition of catalase [14]. O_2_ consumption is reported as a first order rate constant.

Direct consumption of H_2_O_2_ (18 µM) was measured in the same conditions using a H_2_O_2_ electrode (World Precision Instruments, Hertfordshire, UK). In both cases, H_2_O_2_ consumption was reported as a first order rate constant, obtained from the slope of a semi-logarithmic plot of H_2_O_2_ concentration versus time.

### 4.9. Yeast Sensitivity Assays

Acute stress—Yeast cells were grown overnight to mid exponential phase (OD_600_ 1.0). Aliquots of 100 µL were taken from each culture, diluted to 10^−4^ and 100 µL was plated on agar plates. Cells were then treated with 0.5 mM H_2_O_2_ for 5, 15, 30, 45 and 60 min on an orbital shaker at 28 °C. After each incubation time, 100 µL aliquot was taken from each condition, diluted to 10^−4^ and 100 µL was plated on agar plates. As a control for maximum viability, aliquots of 100 µL cells without treatment were also plated. Agar plates were then incubated for 3 days at 28 °C, till visible growth was observed and colonies were then counted. Results are expressed as percentage of the time 0 (non-treated cells) colony number.

Chronic stress—Growth assays were performed on solid YNB medium, supplemented with 2% (*w*/*v*) glucose. Solid YNB medium with 1.5 mM H_2_O_2_ was freshly prepared at the time of inoculation for oxidative stress experiments. Yeast strains were grown in liquid YNB medium, with orbital shaking, at 28 °C up to OD_600_ ≈ 1.0 corresponding to 1 × 10^7^ cells/mL (Gallenkamp Cooled Orbital Incubator). Cells were harvested by centrifugation (4000× *g*; 10 min; 24 °C) (Centrifuge 5810 R Eppendorf, Wien, Austria), washed in sterile distilled water, and re-suspended to OD_600_ ≈ 10. Multi-well plates were prepared with serial 10-fold dilutions of the original concentrated culture up to 10^−5^, 3 µL suspensions were spotted with replica platter for 96-well plates device on plates containing YNB solid medium with and without H_2_O_2_ and incubated at 28 °C. Differences in growth phenotypes of yeast strains were recorded after 1 and 2 weeks of incubation.

### 4.10. Preparation of Cell Lysates for Colorimetric Assays

For antioxidant measurements, yeast strains cultivated to OD_600_ 1.0, were harvested by centrifugation at 3000× *g* for 5 min, and washed with dH_2_O and dry pellets were stored at −80 °C until analysis. Dry pellet was dissolved in phosphate buffered saline (PBS) and disrupted mechanically by vigorous agitation with acid washed glass beads for 7 one-minute intervals with cooling intervals between each agitation cycle. After disruption, cell lysates were cleared by centrifugation at 16,000× *g*, 15 min, RT and the supernatants were used for assays. Prior to performing the assays, protein concentration of cell lysates was determined according to Bradford, using bovine serum albumin as a standard [75].

### 4.11. Catalase Activity Analysis

The catalase activity was measured by modified method of Goth [76]. This method is based on the measurement of H_2_O_2_ degradation in cell lysate, which occurs mostly by catalase activity as it has one of the highest turnover numbers among all the enzymes. For catalase activity assay, 40 μL of supernatant was mixed with 65 mM H_2_O_2_ for the start of the reaction. Different dilutions of hydrogen peroxide (0–75 mM) were used for standards. The reaction was stopped after 5 min by addition of 100 μL of 200 mM ammonium molybdate and color development was measured spectrophotometrically in a plate reader at 405 nm (Shimadzu UV-1601, Kyoto, Japan). One unit of catalase activity is defined as the amount of enzyme needed for degradation of 1 μmol of H_2_O_2_/min at 25 °C. Catalase activity was expressed as units of catalase per milligram of proteins in cell lysate (U·mg^−1^).

### 4.12. Determination of GSH Levels

The intracellular GSH content was measured by modification of the protocol described by Tietze [77]. Briefly, samples were diluted to 0.03 mg/mL protein and 150 μL of each was used for the assay. Reduced glutathione in serial dilutions (0–20 mg/mL) was used as a standard. Reaction was started by addition of freshly prepared reaction mix: 1.8 mM 5,5-dithio-bis-2-nitrobenzoic acid, 0.4 U GSH reductase, and 0.6 mM NADPH in phosphate buffer (100 mM NaH_2_PO_4_, 5 mM EDTA pH 7.4). The formation of 2-nitro-5-thiobenzoic acid was monitored spectrophotometrically in a plate reader at 405 nm (Shimadzu UV-1601, Kyoto, Japan). GSH concentration in cell lysates was expressed as µM of GSH per milligram of total protein (nmol·mg^−1^).

### 4.13. Intracellular ROS Analysis

To evaluate H_2_O_2_ influx, oxidation kinetics of non-fluorescent probe 2’,7’-dichlorodihydrofluorescein diacetate (DCFH-DA, Fluka, Saint Louis, MO, USA) to fluorescent 2,7-dichlorofluorescein was measured within 60 min from H_2_O_2_ administration. Briefly, yeast cells at OD_600_ 1.0 were pre-incubated with 100 μM DCFH-DA at 28 °C for 60 min, centrifuged at 3000× *g* for 5 min. Pellet was washed once with dH_2_O, centrifuged at 3000× *g* for 5 min, and resuspended in media. Cells were then seeded in white microwell plates and treated with 10 mM H_2_O_2_. Fluorescence was measured before treatment and every 10 min after treatment for 60 min with a Cary Eclipse Fluorescence Spectrophotometer (Varian, CA, USA) with excitation at 500 nm and emission detection at 530 nm.

### 4.14. Statistical Analysis

All experiments and assays were carried out in triplicate. Mean values were compared using ANOVA followed by unpaired *t*-test. *p*-values < 0.05 were considered significantly different.

## 5. Conclusions

The present study provides experimental evidences for the direct regulation of AQP5 water permeability by phosphorylation at mammalian physiological pH. In addition to triggering AQP5 trafficking and increasing its membrane abundance, AQP5 phosphorylation promotes an increase in water permeability of cell membranes, which may be needed for rapid volume adaptation and for changes in cell shape crucial for cell migration and proliferation as seen in cancer cells.

AQP5 is also shown to participate in cell oxidative cell response after a stress insult, probably in a dual mechanism where AQP5–H_2_O_2_ permeation activates signal transduction cascades that stimulate antioxidants gene expression. While an initial acute stress induces cell sensitivity, under chronic stress conditions similar to tumor cell niches, AQP5 may facilitate metabolic adaptation, cell survival, and resistance. In this context, AQP5 modulation by phosphorylation may represent a novel strategy with potential application in cancer treatment.

## Supplementary Materials

Supplementary materials can be found at www.mdpi.com/1422-0067/17/12/2090/s1.

## Abbreviations:

AQP	Aquaporin
cAMP	Cyclic adenosine monophosphate
GSH	Glutathione
Ea	Activation energy
Pf	Osmotic permeability coefficient
PKA	Protein kinase A
ROS	Reactive oxygen species
