# Supplementary material for: Rat Aquaporin-5 Is pH-Gated Induced by Phosphorylation and Is Implicated in Oxidative Stress
Source: Int J Mol Sci. 2016 Dec 13;17(12):2090. doi: 10.3390/ijms17122090 (PMC5187890; doi:10.3390/ijms17122090)
Supplement: Supplementary file 1 [file ijms-17-02090-s001.pdf]

Supplementary Materials: Rat Aquaporin-5 Is pH-Gated Induced by Phosphorylation and Is Implicated in Oxidative Stress

Claudia Rodrigues, Andreia Filipa Mósca, Ana Paula Martins, Tatiana Nobre, Catarina Prista, Fernando Antunes, Ana Cipak Gasparovic and Graça Soveral

```
         ....|....| ....|....| ....|....| ....|....| ....|....|
                 10         20         30         40         50
rAQP5    MKKEVCSLAF FKAVFAEFLA TLIFVFFGLG SALKWPSALP TILQISIAFG
hAQP5    MKKEVCSVAF LKAVFAEFLA TLIFVFFGLG SALKWPSALP TILQIALAFG
         *******:** :********* ********** ********** *****::***

         ....|....| ....|....| ....|....| ....|....| ....|....|
                 60         70         80         90        100
rAQP5    LAIGTLAQAL GPVSGGHINP AITLALLIGN QISLLRAVFY VAAQLVGAIA
hAQP5    LAIGTLAQAL GPVSGGHINP AITLALLVGN QISLLRAFFY VAAQLVGAIA
         ********** ********** *******:** *******.** **********

         ....|....| ....|....| ....|....| ....|....| ....|....|
                110        120        130        140        150
rAQP5    GAGILYWLAP LNARGNLAVN ALNNNTTPGK AMVVELILTF QLALCIFSST
hAQP5    GAGILYGVAP LNARGNLAVN ALNNNTTQGQ AMVVELILTF QLALCIFAST
         ****** :** ********** ******* *: ********** *******:**

         ....|....| ....|....| ....|....| ....|....| ....|....|
                160        170        180        190        200
rAQP5    DSRRTSPVGS PALSIGLSVT LGHLVGIYFT GCSMNPARSF GPAVVMNRFS
hAQP5    DSRRTSPVGS PALSIGLSVT LGHLVGIYFT GCSMNPARSF GPAVVMNRFS
         ********** ********** ********** ********** **********

         ....|....| ....|....| ....|....| ....|....| ....|....|
                210        220        230        240        250
rAQP5    PSHWVFWVGP IVGAMLAAIL YFYLLFPSSL SLHDRVAVVK GTYEPEEDWE
hAQP5    PAHWVFWVGP IVGAVLAAIL YFYLLFPNSL SLSERVAIIK GTYEPDEDWE
         *:******** ****:***** *******.** ** :***::* *****:****

         ....|....| ....|
                260
rAQP5    DHREERKKTI ELTAH
hAQP5    EQREERKKTM ELTTR
         ::*******: ***::
```

Figure S1. Sequence alignment of the full-length amino acid sequences of *Rattus norvegicus* AQP5 and human AQP5. The alignment was generated with CLUSTAL W (using BioEdit software). The amount of conservation of each amino acid is represented by symbols ('*', exact match in all sequences; ':', conserved substitution; '.', semi-conserved substitution). The two conserved NPA signature motifs are underlined. Ser152, S156, Ser231, Ser233, Thr242, Thr259 and Thr263 (**colored green**) are consensus phosphorylation sites of hAQP5; His173 and Ser183 (**colored yellow**) are the residues involved in the switch between narrow and wide conformations in the gating mechanism of human AQP5 proposed by Janosi et al. (doi:10.1371/journal.pone.0059897).
